# How Do We Assess Energy Availability and RED-S Risk Factors in Para Athletes?

**DOI:** 10.3390/nu14051068

**Published:** 2022-03-03

**Authors:** Kristin L. Jonvik, Birna Vardardottir, Elizabeth Broad

**Affiliations:** 1 Department of Physical Performance, Norwegian School of Sport Sciences, 0806 Oslo, Norway; 2 Faculty of Health Promotion, Sport and Leisure Studies, University of Iceland, 105 Reykjavik, Iceland; biva@hi.is; 3 Independent Researcher, Shoalhaven, NSW 2540, Australia; lizbroadnutrition@gmail.com

**Keywords:** low energy availability, resting metabolic rate, hormones, bone health, assessment

## Abstract

Low energy availability (LEA) is considered to be the underlying cause of a number of maladaptations in athletes, including impaired physiological function, low bone mineral density (BMD), and hormonal dysfunction. This is collectively referred to as ‘Relative Energy Deficiency in Sport’ (RED-S). LEA is calculated through assessment of dietary energy intake (EI), exercise energy expenditure (EEE) and fat-free mass (FFM). The incidence of LEA in Paralympic athletes is relatively unknown; however, there are legitimate concerns that Para athletes may be at even higher risk of LEA than able-bodied athletes. Unfortunately, there are numerous issues with the application of LEA assessment tools and the criterion for diagnosis within the context of a Para population. The calculation of EEE, in particular, is limited by a distinct lack of published data that cover a range of impairments and activities. In addition, for several RED-S-related factors, it is difficult to distinguish whether they are truly related to LEA or a consequence of the athlete’s impairment and medical history. This narrative review outlines deficits and complexities when assessing RED-S and LEA in Para athletes, presents the information that we do have, and provides suggestions for future progress in this important area of sports nutrition.

## 1. Introduction

The number of Paralympic (Para) athletes, defined as athletes with physical, visual or intellectual impairments classified according to the IPC Classification Code (www.paralympic.org, accessed on 3 February 2022), is increasing. In total, 4000–5000 athletes from all over the world compete in 28 different sports at the Summer and Winter Paralympic Games [1]. The awareness of the importance of adequate nutritional status of Para athletes is increasing and more sport nutritionists are finding opportunities to work with this diverse athletic group. Currently, the dietary counselling of Para athletes is mostly based on nutrition recommendations for able-bodied athletes and the general population, since no research has examined the nutritional needs of the Para athlete. Nutritionists are actively seeking knowledge and effective strategies to provide evidence-based nutrition recommendations for both health and performance to Para athletes [2].

When working with such a heterogenous group, it is important to understand the variety of factors that can impact their nutritional status, such as energy expenditure, dietary intake and body composition [3]. The term ‘Relative Energy Deficiency in Sport’ (RED-S) refers to impaired physiological function including, but not limited to, metabolic rate, hormonal (dys)function, bone health, immunity, protein synthesis, and cardiovascular health. These factors are mainly caused by an imbalance between energy intake (EI) and energy expenditure, resulting in low energy availability (LEA) [4]. Although there is very little research available in Para athletes, it has been suggested that individuals with a disability may experience an increased risk of LEA and an altered physiology related to these conditions when compared with able-bodied athletes [5].

Similar to able-bodied athletes, Para athletes face body image-related challenges [6,7,8] that can result in disordered eating behavior and clinical eating disorders. These issues are, along with unawareness of dietary needs and potential problems with sufficient fueling, known risk factors and/or causes of LEA [9].

Persistent LEA can lead to impaired bone health, which will be further magnified in Para athletes who are non-weight bearing, i.e., wheelchair athletes and/or low-impact sports, due to the lack of loading stimulus [10]. Several studies have reported lower than expected EI in Para athletes based on self-reported food records [11,12,13]. Furthermore, recent studies have attempted to estimate EA, by using Metabolic Equivalent of Task (MET) scores to estimate exercise energy expenditure (EEE) in elite wheelchair Para athletes [14,15]. Egger and Flueck [14] reported LEA in 73% of the measured days in female athletes and 30% of the days in male athletes. In contrast, no athlete in the study of Pritchett et al. [15] was found to have LEA according to cut-off values established for able-bodied athletes, but large daily EA fluctuations were apparent, and the athletes were mostly in a light phase of training.

No study in Para athletes has investigated the direct EA by measuring EEE (instead of estimating it) and evaluating it against EI and FFM. Furthermore, in Para athletes, it is difficult to distinguish whether impaired bone health, reduced resting metabolic rate (RMR), low EI and/or impaired hormone status are related to RED-S or due to their impairment [16]. The question to ask is whether we can measure the risk of RED-S in Para athletes with the tools and measures currently available.

The aim of this article is to highlight the main practical and research challenges faced when assessing EA and risk factors for RED-S in Para athletes. This article will cover the issues that need to be understood in order to assess and evaluate LEA and RED-S. We also focus on the accuracy and relevance of existing methods when used in Para athletes. Secondarily, it is important to consider whether LEA/RED-S or the impairment is contributing the apparent symptoms.

## 2. Energy Availability

EA is the energy available for basic physiological functioning once the energy expended during exercise has been subtracted from energy consumed from food. EA is expressed in kcal/kg of fat-free mass (FFM) [9]. Sufficient EA is of high importance for health and athletic performance, whereas LEA hinders it. What is sufficient EA and how low is low EA? Even for able-bodied athletes, the LEA cut-off/threshold of <30 kcal/kg FFM is unclear. This cut-off threshold was initially set by Loucks and Thuma [17] based on research conducted in sedentary females in a clinical setting. Recently, it has been debated whether such a definite cut-off is applicable to all athletes [18], and if so, it would likely differ between male and female athletes [19]. Whether this cut-off could be applied to Para athletes is also uncertain.

Furthermore, EA estimations are subject to certain difficulties and lack of standardized protocols [19,20]. Laboratory and clinical quantification of EA is difficult and challenged due to significant under- or overestimation of EI [21] and/or EEE [22].

**Figure 1:**
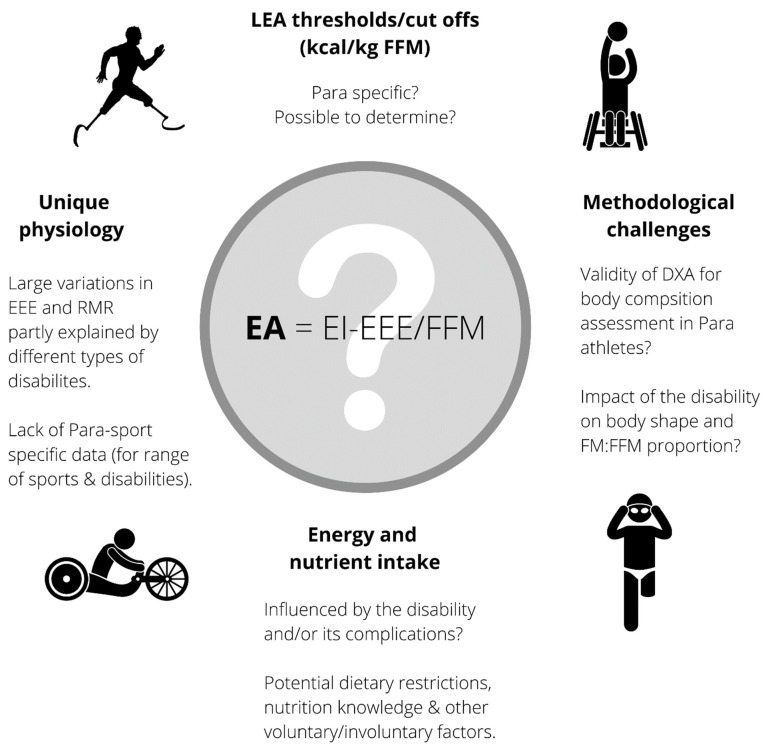
Factors that make the EA formula and low EA (LEA) cut-off/threshold questionable in Para athletes. EA = energy availability (expressed in kcal/kg FFM), EI = energy intake, EEE = exercise energy expenditure, FM = fat mass, FFM = fat-free mass, DXA = dual-energy X-ray absorptiometry, and RMR = resting metabolic rate.

Other factors, such as disability type or severity, may need to be accounted for when it comes to EA assessment in Para athletes [16], and direct measures of EA, including EI, EEE and FFM have only been examined in able-bodied athletes. Figure 1 identifies the main factors that make the EA formula and LEA cut-off/threshold questionable in Para athletes. Recent literature has highlighted that many Para athletes have significant challenges with optimizing EI. When coupled with potentially altered aspects of EEE, EA, and FFM, this could put them at increased risk for LEA and RED-S [5].

### 2.1. Total Energy Intake

RED-S results from insufficient EI in relation to exercise training load. Indeed, several studies have indicated that EI and/or nutrient inadequacies can be a concern for some Para athletes. This includes insufficient total EI and consumption of selected macro- and micronutrients [11,12,13,14]. Potential reasons for low intakes include dietary restrictions, ability to access and/or prepare food, limited nutritional knowledge, impact of changes in fiber content on gastrointestinal function and comfort, and possibly food allergies/intolerances. Furthermore, the use of medications and/or gastrointestinal function may interact with dietary nutrients and interrupt absorption. Insufficient EI can increase the risk for suboptimal macro- and micronutrient status, decreased bone mineral density (BMD), increased susceptibility to illness and injury, weakened oxygen utilization and transport, and a reduced ability to sustain high-intensity training [3]. It is important to remember that assessments of EI in free-living athletes are challenged by the risk of or under- or overreporting, and lack of a single standardized protocol [20]. Egger and Flueck [14] assessed EI of elite wheelchair athletes over seven consecutive days in their pre-season, using weighed and photo-assisted food records. They reported LEA in 73% of all days for female and 30% of male athletes. Female athletes were most at risk for low EI and macronutrient (i.e., protein and carbohydrate) intakes. The dietary method they used is currently considered the best available when assessing EI in free-living athletes [19]. Tracking EI over a full week depicts a training microcycle, and can reduce variability in estimations when compared to shorter periods [23]. The use of photographs has been suggested to be a useful addition to EI assessments in athletes and analysis [21], but still requires a trained researcher to accurately quantify. Lastly, gathering information on any periodized nutritional approach used [24] can explain between-days or training period differences in EI, EA and/or nutrient distribution. Research on EI in Para athletes is still new, and further work is required to elucidate how they differ from able-bodied athletes using weighed 7 day food diaries.

### 2.2. Exercise Energy Expenditure

EEE varies enormously across, and within, Para athlete groups, due to the wide variation in how the impairment impacts the physiological response to exercise (e.g., movement efficiency, muscle mass, types of prosthetic tools, sporting equipment, and physique traits), the clinical presentation (e.g., level of spinal cord injury, location of amputation, degree of severity of cerebral palsy), level of training (elite vs. sub-elite) and sex. For instance, a 2-fold variation in EEE was found across 5 elite subjects in a 25 km wheelchair racing time trial [25]. Importantly, EEE in Para athletes is highly understudied, and the data that exist are predominantly in wheelchair athletes. The accurate estimation of daily energy expenditure is costly and limited primarily to research environments. Currently, no study has used Doubly Labelled Water (DLW) to calculate the total energy expenditure (TEE) in Para athletes. More portable and cheaper tools, such as wearable devices, have limitations in their accuracy and applicability across a range of activities and body types in able-bodied individuals [26,27,28,29]. For some ambulatory Para athletes, smart watches and other common tools, may be applicable for providing a ‘ball park’ estimate of TEE. Their accuracy for athletes who mobilize and/or train in a wheelchair, or who have muscle atrophy, unstable gait, or substantially different limbs is uncertain as the assumptions built into the algorithms by which they operate may be invalid. Nightingale et al. [30] provide a comprehensive assessment of the accuracy of such tools for wheelchair users; however, there is minimal information on their accuracy across other Para athletes.

A compendium of MET scores for the use of a manual wheelchair during different activities has been developed [31]. This compendium is based on 11 studies, where only 4 studies included Para athletes [16]. These data are primarily based on older studies with small sample sizes using older style racing equipment, which is not as aerodynamic or lightweight as that used today. To accurately estimate the MET score of an exercise, there is a need to fully understand the factors that can impact total and exercise energy expenditure in Para athletes [14]. Currently, there are no data or guidance as to how best to adjust for the gait instabilities that occurs in ambulatory athletes with lower-limb impairments. Movement inefficiency (e.g., in lower-limb amputations) can increase the metabolic demand and, hence, EEE [32]; however, the impact varies according to the type of impairment, use of prosthetics, and the movement patterns required of the sport. Furthermore, MET scores can be difficult to apply to ‘stop–start’ sports, where athletes quickly change pace and direction (such as team sports). Estimation of EEE relies on an accurate estimate of RMR and the MET score, or direct measures of EEE across individuals [14]. In research settings, portable indirect calorimetry devices can be used for certain types of exercise [33]. This instrumentation can provide valuable information to validate MET scores and EEE for different Para athlete impairments and type of sport activities.

### 2.3. Fat-Free Mass

In order to estimate RMR, a measure of FFM is needed, which is typically measured by dual-energy X-ray absorptiometry (DXA). However, the validity of DXA as a tool to assess FFM in Para athletes is still unknown, since there are large variation in body shapes, stature, and distribution of FFM between body compartments. However, DXA scans can be used for segmental body composition measurements in order to compare body composition of Para athletes to able-bodied athletes for similar sports disciplines [34,35]. Currently, it is questionable whether using FFM for the estimation of EA applies to Para athletes, when the cut-off values for able-bodied athletes are used. An athlete with less total and/or active FFM, will likely have both a different EI and EEE than their able-bodied counterparts, thus, impacting the estimation of EA [3]. Anthropometry data from large groups of Para athletes are necessary to generate reference values for FFM and EA cut-offs, which is a challenge due to the heterogeneity of this group.

## 3. Other Factors of RED-S

### 3.1. Questionnaires

The LEAF-Q questionnaire [36] assesses selected physiological outcomes of LEA, including injuries, gastrointestinal and reproductive function, in able-bodied female athletes. Since its development, our understanding of LEA/RED-S and its effects on different body systems in male and female athletes has expanded. Therefore, it has been questioned whether the initial version of LEAF-Q is valid in all athlete populations [37]. The LEAF-Q must be used with other questionnaires to screen for body image issues, eating disorders and other potential challenges [36,37]. Although LEAF-Q is widely used for screening of RED-S risk it has only been validated for use in adult endurance athletes, and its relevance for Para athletes warrants further research. Outlined below are several Para-specific aspects that may need to be accounted for to make screening of RED-S risk fully applicable to this group.

### 3.2. Body Image and Eating Disorders

Living with a disability often means dealing with and overcoming physiological and psychological barriers, and sometimes relying on wheelchairs, prostheses or other assistive devices in daily lives. Some individuals describe their body image as evolving, i.e., after an injury causing the impairment occurs, or a diagnosis is given, as they learn to live and train in a body that functions differently than before the injury [6]. Conversely, sport participation improves physical ability, and self-confidence is often greatly increased for those reasons [38]. Whether negative body image and risk of eating disorders are more related to their disability, athletic status and pressure, or a combination of both remains to be elucidated. In their study on United States Para athletes, Brook et al. [8] reported that as many as 61.5% were attempting to change their body composition or weight for improved athletic performance. Additionally, 18.5% had an elevated dietary restraint subscale score and 32.4% had elevated pathologic behavior subscale scores, as assessed by the Eating Disorder Examination Questionnaire (EDE-Q). A positive correlation between body image scores and ideal eating behavior was found in Japanese Para athletes [7]. This provides evidence that improved nutritional support, tailored to the needs of Para athletes, might result in improved body image and mental well-being. However, the prevalence and main consequences of negative body image and eating disorders in Para athletes warrant further research.

### 3.3. Bone Health

Exercise-related factors most important for BMD include weight-bearing exercise, intensity and strength training which protects bone [39]. Some Para athletes, particularly those using wheelchairs, lack this mechanical bone loading that may put them at increased risk of low BMD and associated complications [40]. Reduced BMD increases the risk of stress fractures and bone injuries in athletes, which can limit the athlete’s ability to train or compete [41]. This is of particular concern in athletes with SCI who experience a substantial loss of BMD immediately post injury, and hence have a high incidence of reduced bone density [42]. As much as 50% of the fractures in individuals with SCI result in secondary complications such as infections, pressure ulcers, prolonged healing time, autonomic dysreflexia, and greater muscle spasticity [43]. While the athlete may not ‘feel’ the pain of a bone injury in insensate limbs, their body may show distinct signs of this injury. Examples of that include increased spasm, or in athletes with high-level SCI, autonomic dysreflexia which is a potentially fatal condition of high blood pressure in response to a ‘noxious stimulus’. Additionally, the inflammatory reaction to a bone fracture may result in cellulitis, skin breakdown and potentially amputation if left unmanaged [42]. In other Para athlete groups, reduced BMD may be present in limbs that experience reduced gravitational loading (such as a limb with a prosthesis or where significant muscle atrophy is present) or may be the cause of their impairment (such as osteogenesis imperfecta).

DXA is the gold standard for BMD assessments; however, it uses able-bodied reference data to determine age- and gender-matched stratification of BMD (i.e., Z-scores) and clinical cut-offs for osteopenia and osteoporosis. The validity of these reference data for the Para athlete is uncertain, and the measurement of BMD itself is a challenge. Para athletes can have a deformity, contracture, heterotopic ossification, spasticity or other factors that prevent optimal positioning on the scanning bed, thus, limiting the accuracy of analysis. As metal is often an issue when scanning hip and spine of Para athletes, monitoring BMD of other sites, such as proximal tibia, distal femur or distal radius in wheelchair-bound athletes, may be pertinent when assessing change over time [42]. However, we currently lack reference values to evaluate the BMD of these sites in Para athletes, which limits the interpretation of results.

In addition to the lack of mechanical loading, low BMD can also be directly related to RED-S and chronic LEA [4]. When assessing BMD values in relation to LEA in Para athletes, it must be acknowledged that low BMD could be related to the impairment, regardless of diet quality or EI [15]. As such, a single assessment of BMD may be an inappropriate diagnostic criterion for assessing the risk of LEA in Para athletes. Therefore, research on EA and bone health in Para athletes has been identified by the IOC as a focus area for future research [44].

### 3.4. Resting Metabolic Rate

A reduced RMR has been identified as a potential biomarker of LEA/RED-S [4] in able-bodied athletes. However, in Para athletes, RMR might deviate from the able-bodied population as a result of factors related to the impairment. As such, it is difficult to establish whether RMR is related to LEA or the impairment. Individuals with SCI can have a reduced measured RMR compared with the predicted RMR from equations for the able-bodied population due to their reduced FFM [30]. Conversely, competitive wheelchair athletes, on the other hand, have shown higher predicted RMR, as they exhibit a higher RMR/FFM ratio [45].

RMR can be measured by indirect calorimetry in the laboratory, but in the field RMR is frequently estimated. Several equations for estimating RMR require only weight, height and age, such as the formula by Harris and Benedict [46]. These prediction equations have not been validated in Para athletes. Factors such as potential problems with acquiring accurate height and weight measurements, and different ratios of FFM to FM present in Para athletes compared to the population on which the formulas were developed likely make them inappropriate for use. The Cunningham equation [47] is most frequently used to estimate RMR in athletes and has the best precision in wheelchair athletes [45,48]. However, this equation requires access to an accurate method of measuring FFM, (e.g., dual-energy x-ray absorptiometry (DXA) or a BodPod^TM^) [49], There are also FFM-based formulas validated specifically on SCI individuals, such as Chun et al. [50] and Nightingale and Gorgey [51]. Although the population they studied wre not athletes, these formulas can serve as good alternatives in wheelchair athletes [26]. For Para track and field athletes, Juzwiak et al. [52] reported that the Owen [53,54] and Mifflin [55] equations showed the best prediction of RMR. A ratio between measured and predicted RMR of <0.9 has been suggested as a marker for LEA [56]. However, this threshold value was originally determined using the Harris and Benedict equation, and as such does not account for FFM. Whether this threshold applies to Para athletes remains unclear.

### 3.5. Hormones

For female athletes, menstrual disturbances, including hypothalamic amenorrhea or absence of menstruation, are one of the first red flags of LEA/RED-S, and has been well described in the literature [4,57]. In a recent study on female Para athletes [15], menstrual dysfunctions were reported in 4 of 9 (44%) females but the majority (67%) reported using oral contraceptives (OCs). Athletes may use OCs for various reasons, but their use makes menstrual function assessment impossible due to effects of exogenous hormones. For female Para athletes, disabilities and the injuries underpinning them may affect the hypothalamic–pituitary-gonadal axis (HPGA), and impact menstrual function and/or reproductive health [5].

It has been suggested that a similar disruption of the HPGA may occur due to LEA and RED-S in male athletes [58]. Research shows that male athletes with or at risk of RED-S have lower testosterone compared to those with adequate EA [59]. Pritchett et al. [15] reported that all Para male athletes in their study had low testosterone (9–16.5 nmol·L^−1^) and 67% had clinically low levels (<9 nmol·L^−1^). Low testosterone is common in men with SCI [60], potentially making it difficult to isolate the main cause.

## 4. How Can We Assess LEA and RED-S in the Future?

Assessing LEA and RED-S in the Para athlete still requires further research validation. Two primary areas of focus are highlighted below:

1. Support the individual athlete by embedding experienced sports nutrition, medical and exercise science practitioners into Para sports programs. These practitioners require time to develop a deep understanding of each athlete, their impairment, sport and medical history. This will improve their ability to observe factors that may raise ‘red flags’ and understand the best assessment procedures to implement. Where feasible, Para athletes should be screened early on in their sporting careers for factors such as menstrual (dys)function (female), hormonal status, BMD and body composition, dietary intake and eating behavior, and RMR measured. If concerns regarding LEA/RED-S arise, having comparative data over time will aid in the assessment relative to the current issues and the impairment, sport and medical history of the athlete. The importance of having skilled practitioners in Para sport programs cannot be overstated, since these individuals will be the first to identify a problem. Other factors to consider are potential changes in mood state, performance, immune function, sleep, training capacity, recovery and general well-being.
2. Connect research institutions and funding sources with Para sports, athletes and practitioners to create opportunities for the collection, analyses and publishing of more physiological data on Para athletes. This includes, but is not limited to:
  (a) The expansion of EEE measurements across all Para sports and disciplines in a meaningful and comparative manner. For example, more research needs to be reported in kcal/kg/h rather than just watts or kcal/kJ.
  (b) Assessment of dietary intake of Para athletes in free-living situations combined with assessments of measured energy expenditure (either via DLW, or a combination of RMR and EEE measures using indirect calorimetry).
  (c) Best-practice protocols for BMD assessments and normative ranges in Para athletes with different disabilities.
  (d) Identify factors associated with negative body image and disordered eating behavior that are specific to Para athletes, including tools and programs to improve them.
  (e) Incidence, cause and potential treatment of disrupted reproductive hormones in male and female Para athletes.

## 5. Conclusions

It has been suggested that Para athletes are at greater risk of LEA and RED-S compared to their able-bodied counterparts, but it is difficult to determine whether performance decrements in Para athletes are due to RED-S or the athlete’s impairment. Research is needed to develop tools, assessment measures and reference ranges commonly used in able-bodied athletes that are appropriate for the Para population. Future work should focus on embedding qualified nutrition, medical, and exercise practitioners in Para sport programs to support the individual athlete. Lastly, research on Para athletes needs to be expanded to provide the data and understanding required for evidence-based guidelines for athletes across a range of disabilities.
